# Supplementary material for: Health professional and patient views of a novel prognostic test for melanoma: A theoretically informed qualitative study
Source: PLoS One. 2022 Apr 4;17(4):e0265048. doi: 10.1371/journal.pone.0265048 (PMC8979436; doi:10.1371/journal.pone.0265048)
Supplement: S1 File — (DOCX) [file pone.0265048.s001.docx]

**AMLo Topic guide`1 - Patients and carers**

- Time since diagnosis
- Experience of diagnosis and care
  - What told - risks
  - Issues/what could have been improved
  - FU policy
- New test
  - General views (questions)
  - Understanding
  - Trust
  - Compulsory?
  - Perspectives on impact on
    - Patients – including any difference it would have made in their own case
    - Changes to FU?
    - Families
    - Clinical team
    - Others in diagnostic and care pathway
    - Decision making
  - Implementation – barriers; facilitators
