# Supplementary material for: Health professional and patient views of a novel prognostic test for melanoma: A theoretically informed qualitative study
Source: PLoS One. 2022 Apr 4;17(4):e0265048. doi: 10.1371/journal.pone.0265048 (PMC8979436; doi:10.1371/journal.pone.0265048)
Supplement: S2 File — (DOCX) [file pone.0265048.s002.docx]

**AMLo Topic guide - Professionals**

- Current diagnostic pathway*
  - Issues
- Current care pathway*
  - Issues
- Follow-up
- New test
  - General views (and questions)
  - Pros and cons
  - Impact on practice
    - Time
    - Cost
    - Communication
    - Decision making
  - Impact on others in the diagnostic/care pathway
  - Trust
  - Implementation – barriers; facilitators
- Training
